# Effects of Exergame Training on Executive Function and Heart Rate Variability in Middle‐Aged and Older Adults: A Randomized Controlled Study

**DOI:** 10.1002/ejsc.12249

**Published:** 2025-01-16

**Authors:** Tzu‐Cheng Yu, Pei‐Tzu Wu, Wen‐Lan Wu, Yu‐Kai Chang, Che‐Hsien Chiang, I‐Hua Chu

**Affiliations:** ^1^ Ph.D. Program in Biomedical Engineering College of Medicine Kaohsiung Medical University Kaohsiung Taiwan; ^2^ Doctor of Physical Therapy Southen California University of Health Sciences Whittier California USA; ^3^ Department of Sports Medicine College of Medicine Kaohsiung Medical University Kaohsiung Taiwan; ^4^ Department of Physical Education and Sport Sciences National Taiwan Normal University Taipei Taiwan; ^5^ Institute for Research Excellence in Learning Science National Taiwan Normal University Taipei Taiwan; ^6^ Department of Medical Research Kaohsiung Medical University Hospital Kaohsiung Taiwan

**Keywords:** behavior, cognition, cardiovascular/cardiorespiratory, exercise, fitness, physiology, psychology, sports medicine

## Abstract

Exergame has become widely popular and offers great levels of cognitive demands, thus may facilitate cognitive benefits. In addition, researchers have proposed that cardiac autonomic function, assessed via heart rate variability (HRV), is associated with cognitive executive function. However, few exergame training studies have investigated this relationship. This study aimed to investigate the effects of 10‐week exergame training on executive function and HRV in middle‐aged and older adults. Ninety‐one participants were randomly assigned to either an exergame training group (63.73 ± 4.48 years) or a control group (62.46 ± 4.77 years). The training program was 50 min per session, twice per week for 10 weeks. The control group was instructed to maintain their usual lifestyle. All participants completed questionnaires and received assessments of executive functions and electrocardiography at baseline and postintervention. Results revealed significant group‐by‐time interaction effects for the three indices of the digit span test and the total initiation time of the Tower of London task with better postintervention performances achieved by the exergame group. The 6 min walk test also improved significantly in the exergame group but not in the control group. No significant change in HRV was observed for both groups. There were significant correlations between HRV and digit span test scores. Our results suggest that the 10‐week exergame training program was effective in improving executive functions of working memory, inhibitory control, and planning ability as well as aerobic fitness in middle‐aged and older adults. Moreover, HRV was associated with improved executive function.

1

**Summary**

- A 10‐week exergame training program was associated with enhanced executive functions of working memory, inhibitory control, and planning ability.
- Exergame training improved aerobic fitness and blood pressure in middle‐aged and older adults.
- HRV was associated with the improvements in working memory.

**Abbreviations**
BMI: Body mass index
CON group: Control group
DSB: Digit span backward
DSF: Digit span forward
DST: Digit span test
ECG: Electrocardiogram
EX group: Exergame group
GLEQ: Godin Leisure‐time Exercise Questionnaire
HRV: Heart rate variability
MMSE: Mini–Mental State Examination
RMSSD: Root mean square of successive RR interval differences
SDNN: Standard deviation of normal‐to‐normal intervals
TIT: Total initiation time
TOL: Tower of London
6MWT: Six‐minute walk test

## 2 Background

Exergaming is a novel form of exercise that allows individuals to perform exercise with fewer environmental barriers (e.g., locations and climate) and was shown to be an enjoyable mode of exercise that promotes long‐term activity participation in older adults (Moholdt et al. 2017). Players play exergames through body movements, such as jumping, squatting, running in place, and side‐stepping (Chaput et al. 2015; Gao et al. 2015; Su et al. 2015). Studies have shown that players were able to achieve light‐to‐moderate intensity of exercise using the Xbox Kinect (Chaput et al. 2015; Maddison et al. 2012). This exergame was also shown to improve aerobic endurance and leg muscle strength in middle‐aged and older adults (Yu et al. 2020). Exercise training that improves aerobic endurance is essential for the prevention, treatment, and control of high blood pressure (Pescatello et al. 2004), a major risk factor for cardiovascular disease and the leading cause of death globally (Aune et al. 2021). Therefore, exergame training can also be beneficial for the management of blood pressure.

Exergaming also offers great levels of cognitive demands in response to a complex and unpredictable environment (Torre et al. 2022). Therefore, exergaming, defined as “physical exercise interactively combined with cognitive stimulation in a virtual environment” (van Santen et al. 2018), may also facilitate cognitive benefits. Several studies have demonstrated improved cognitive function after an acute bout of exergaming session (Best 2012) as well as long‐term exergame training in both youth and older adults (Barcelos et al. 2015; Best 2013; Anderson‐Hanley et al. 2012). A recent review and meta‐analysis investigated the dose–response relationships of specific exercise and training variables of exergame training on cognitive function in middle‐aged (45–65 years) and older adults (Manser, Herold, and de Bruin 2024). The results showed that exergame training was effective in improving global cognition, complex attention, and executive functions. In addition, exergame training at moderate intensity was favorable to improve executive functions (Manser, Herold, and de Bruin 2024).

Executive functions are higher‐order mental capacities that allow one to adapt to new situations and achieve goals. They include multiple functions such as decision‐making, problem solving, inhibition, working memory, shifting, and planning (Gillen 2008; Diamond 2013). Working memory is required when translating instructions and incorporating new information into action plans (e.g., holding information to complete tasks in the game) (Diamond 2013). Planning ability is part of core cognitive processes that involve the use of strategies with multiple steps to achieve specific goals (e.g., winning the game and getting higher scores) (Tomporowski et al. 2015). Therefore, working memory and planning ability may be stimulated while playing exergames. A number of studies have demonstrated positive effects of exergame training on working memory in healthy older adults (Zhao et al. 2022; Adcock et al. 2019; Moret, Nucci, and Campana 2022) and older adults with hypertension (Hou et al. 2023) and mild cognitive impairment (Liu et al. 2022). On the other hand, it is unclear how exergame impacts planning ability in middle‐aged and older adults. The effect of exergame training on this executive function has not been investigated previously in these populations (Torre et al. 2022; Manser, Herold, and de Bruin 2024; Stojan et al. 2019).

Cardiac autonomic function has been linked to executive function. Individuals with higher heart rate variability (HRV), an indicator of cardiac autonomic regulation, demonstrate greater executive function (Zeki Al Hazzouri et al. 2014; Jennings et al. 2015). Results from a large meta‐analysis (*N* = 14,347) also reported that greater HRV was related to better executive functions, and this association was stronger in older adults (Holzman et al. 2017). These findings support the proposed “neuro‐visceral integration model” that links executive functions to cardiac autonomic regulation (Thayer et al. 2009) and suggest the use of HRV indices as biomarkers of executive functions (Holzman et al. 2017). Research conducted in both young (mean age 19.1 years) and older adults (mean age 70.7 years) reported increased HRV and improved executive functions after aerobic exercise training and suggested a link However, this relationship, has not been examined extensively in the exergame literature. Only one study to date has examined the effect of exergame training on HRV and executive functions. The results suggest that exergame training was an effective training strategy to improve HRV in older adults and that HRV was associated with executive functions (P. Eggenberger et al. 2020).

The purpose of this study was to investigate the effect of a 10‐week exergame training on executive functions of working memory and planning ability and HRV in middle‐aged and older adults. We hypothesized that exergame training would improve working memory, planning ability, and HRV and that there would be correlations between these executive functions and HRV.

## 3 Methods

### 3.1 Participants

Volunteers were recruited from the university and surrounding communities via word‐of‐mouth, emails, and posters. The inclusion criteria were (1) age ≧ 55 years; (2) 18.5 ≦ BMI ≦ 29.9 (body mass index); and (3) Mini–Mental State Examination (MMSE) ≧ 25. Volunteers were excluded if they (1) had musculoskeletal problems that may affect physical fitness tests and exercise training (e.g., joint pain or inflammation); (2) were unable to walk independently (e.g., using a walker); (3) had cardiovascular disease, diabetes, pulmonary disease or kidney disease; or (4) had any physical contraindications to perform exercise.

The sample size of 52 participants (26 in each group) was calculated with an alpha level of 0.05, a power of 0.80, and an effect size of 0.20 using the G*POWER (Faul et al. 2007). The effect size was calculated using data from our pilot study. All participants were informed about the purpose, procedures, and precautions of the study before they were asked to sign and return the written Clinical Trial Informed Consent Form approved by the Kaohsiung Medical University Institutional Review Board (KMUHIRB‐SV(II)‐20150055) and were performed according to the Declaration of Helsinki.

### 3.2 Physical Activity Level

Physical activity level was assessed to monitor levels of physical activity using the Godin Leisure‐time Exercise Questionnaire (GLEQ) (Godin et al. 1985). Weekly strenuous (e.g., running), moderate (e.g., fast walking), and light (e.g., yoga) physical activities were recorded. A weekly leisure activity score was calculated using the following formula:

(1) Weeklyleisureactivity=(9×Strenuous)+(5×Moderate)+(3×Light)

### 3.3 Aerobic Endurance

The six‐minute walk test (6MWT) was used to assess aerobic endurance. The test was performed along an undisturbed 20 m flat corridor with the starting line and the turnaround point marked with cones. The participant was instructed to walk back and forth along the corridor as fast as possible for 6 min. The walking distance (in meters) covered in 6 min was recorded (Heyward et al. 2014).

### 3.4 Cognitive Function Assessments

The mini–mental state examination is a 30‐item questionnaire used to assess cognitive impairment. The questionnaire is divided into eight aspects, namely temporal orientation, spatial orientation, registration, attention and calculation, remote memory, naming, repeat, stage command, writing, reading, and copy, with a total of 30 points. The higher the score, the better the cognitive ability. To ensure that the participants understand how to perform exergames, those who scored under 25 on the scale were excluded from the study (O'Connor et al. 1989).

The digit span test was used to evaluate working memory (Miller 1994; Gardner 1981; Kaufman et al. 1999; Wechsler 1944). The test consists of a random series of digits. The participants will hear a sequence of digits and are asked to verbally repeat the sequence immediately in either the order presented (forward span, DSF) or in reverse order (backward span, DSB). If the participant repeats the sequence correctly, then a longer sequence is presented. The test ends when the participant repeated the sequence incorrectly on three occasions at a span length. The scores for the forward span (16 points in total), backward span (14 points in total), and total score (sum of both scores, DST; 30 points in total) were recorded, with higher scores indicating better cognitive functions.

The Tower of London (TOL) task is a neuropsychological assessment that involves planning, strategies, procedural knowledge, and declarative knowledge (Tomporowski et al. 2015; Strauss, Sherman, and Spreen 2006; Culbertson 2005). In the task, there are two identical sets of boards and colored balls (i.e., red, green, and blue balls). One board belongs to the experimenter and the other one belongs to the participant. Each board has three vertical pegs with different heights. All three balls can be placed on the tallest peg, two can be placed on the middle peg, and only one ball can be placed on the shortest peg. There are 10 questions in the TOL task. The experimenter arranged the three balls on his own board for each question. The participants were instructed to move the balls from the start position to the question position using the minimum number of moves possible. Four performance scores for the TOL task are described as follows.

1. Total Move Score: The sum of move differences between the actual ball moves and the standard moves for each question.
2. Total Initiation Time: The sum of the duration of time before the first move for each question.
3. Total Execution Time: The sum of the time used to complete each question.
4. Total Time: The sum of the total initial time and the total execution time.

### 3.5 Heart Rate Variability

Heart rate variability (HRV) was derived from continuous heart rate recording, at a sampling rate of 1024 Hz, using an electrocardiogram (ECG) system (ProComp Infiniti System, Thought Technology Ltd., Montreal, Quebec, Canada). The resting ECG was recorded for 10 min with the participants rested in a sitting position. The last 5 min of the ECG data were analyzed for HRV using the Chart 5 for the Windows (ADinstruments, Australia) system. Four HRV indices were recorded and used in data analysis, including two time‐domain indices (standard deviation of normal‐to‐normal intervals, SDNN and root mean square of successive RR interval differences, RMSSD) and two frequency‐domain indices (total power, TP and relative power of the high‐frequency band (0.15–0.4 Hz) in normal units, HFnu).

### 3.6 Exergames and Traning Protocols

The two exergame software (Kinect Sports and Kinect Adventures) were used in the training session. These games were chosen as they were commonly provided with the console when purchased and were among the most popular games when the Xbox 360 Kinect was released. When playing these games, participants need to hold information for different games to complete various tasks as well as plan strategically in order to earn more points/higher scores in each game. As such, working memory and planning ability may be stimulated while playing exergames. During weeks 1, 3, 5, 7, and 9, participants performed two exergame items (15 min each) of the Kinect Sports (e.g., boxing and bowling) in each training session. During weeks 2, 4, 6, 8, and 10, participants performed all five exergame items of the Kinect Adventures, 3 min per item, and repeated them twice. The durations for the exergames varied due to the different designs and settings for each exergame. All exergame training sessions were conducted in the laboratory and supervised by the experimenters.

### 3.7 Procedures

The eligible participants were randomly assigned to either the exergame group (EX group and *n* = 46) or the control group (CON group and *n* = 45) using simple randomization with a random sequence generated by the computer software. The EX group underwent exergame training twice per week, 50 min per session, for 10 weeks (Stojan et al. 2019). The training session was conducted individually and supervised by research staff. Each exergame training session consisted of 10 min of warm‐up, 30 min of exergame, and 10 min of cool‐down. The participants in the CON group were instructed to maintain their lifestyle. The posttest was conducted after 10 weeks and was scheduled at least 72 h after the last training session to avoid the short‐term effects of exercise training.

Participant's blood pressure (OMRON HEM‐7200, OMRON HEALTHCARE Co. Ltd., Kyoto, Japan) and heart rate (Mio Fuse, JoiiUp Technology Inc., Taipei, Taiwan) (Wang et al. 2017) were measured at pretest and posttest and each training session. If the participant's systolic blood pressure≧ 160 mmHg or diastolic blood pressure≧ 110 mmHg at rest, the exercise testing or training was postponed. The training intensity was prescribed at moderate intensity (40%–60% of the heart rate reserve (difference between the peak heart rate and the resting heart rate)) (Stojan et al. 2019; Heyward et al. 2014). During each training session, participants were provided verbal cues to ensure their heart rate stayed within the prescribed heart rate range.

### 3.8 Statistical Analysis

All data analyses were performed using the Statistical Package for the Social Sciences Version 22.0 (SPSS, IBM Corporation, Chicago, IL, USA). Data were inspected visually and statistically for normality (Shapiro–Wilk's test and *p* > 0.05), and all variables except for three HRV indices (SDNN, RMSSD, and TP) were normally distributed. The three HRV indices were transformed into their natural logarithm (Ln) to permit parametric statistical analysis. The independent *t*‐test and the χ^2^ test were performed to examine any difference between groups at baseline. To examine the effect of exergame training on executive function and HRV, mixed model analysis of variance (ANOVA) with the between‐group variable (EX vs. CON) and within‐group variable (pretest vs. posttest) were performed for all indices of the digit span test and the TOL task and for all indices of HRV. Any significant effect was followed by post hoc comparisons with Bonferroni corrections. Partial eta squared (η^2^) was used as an indicator of effect size. Cohen's *d* was calculated for post hoc comparisons. Pearson’s product‐moment correlations were performed to examine the relationships between the changes in executive function test scores and changes in HRV. The significance level was set at 0.05.

## 4 Results

### 4.1 Participants

**FIGURE 1:**
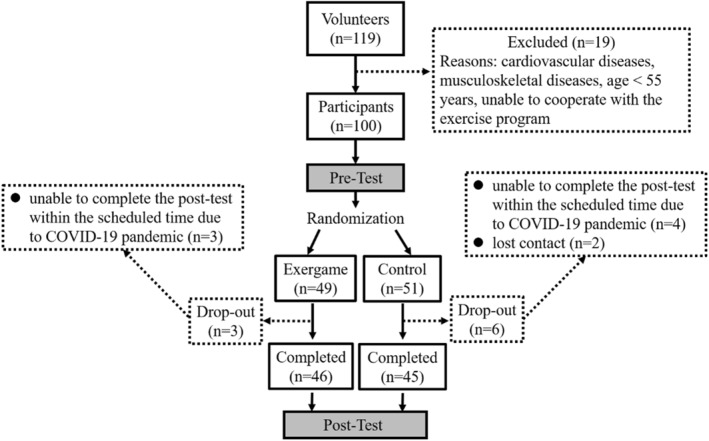
The flowchart of the study.

A total of 91 participants (75 females (82.50%)) completed the study (Figure 1). At baseline, no significant difference in participant characteristics was observed between EX and CON groups (Table 1). The attendance rate of the exergame training program was 77.75%. The duration (*p* < 0.001 and *d* = 1.09), frequency (*p* < 0.001 and *d* = 1.15), and intensity (*p* < 0.001 and *d* = 1.22) of physical activity increased significantly in the EX group at posttest (Table 1), whereas no significant change was observed for the CON group. The results of the 6 min walk test improved significantly in the EX group (*p* = 0.002 and *d* = 0.50) but not in the CON group. Systolic blood pressure decreased significantly in both the EX group (*p* = 0.002 and *d* = 0.49) and the CON group (*p* = 0.033 and *d* = 0.33), and diastolic blood pressure decreased significantly only in the EX group (*p* = 0.001 and *d* = 0.53).

**TABLE 1:** Participants' characteristics.

	Exergame group (*n* = 46)		Control group (*n* = 45)		Time × group	
	Pre	Post	Pre	Post	*p*	η2p
Age (years)	63.73 ± 4.48	(Range: 55–76)	62.46 ± 4.77	(Range: 55–72)	—	—
Gender M: F (n)	8:38		8:37		—	—
BMI (kg/m2)	22.63 ± 2.50	22.58 ± 2.42	22.82 ± 2.54	22.79 ± 2.48	0.830	0.001
PBF (%)	28.56 ± 7.79	28.75 ± 7.49	29.82 ± 7.24	29.62 ± 7.32	0.385	0.008
Duration (min/wk)	300.65 ± 274.23	420.21 ± 266.23^a^	264.77 ± 243.63	257.88 ± 223.14	< 0.001	0.235
Frequency (times/wk)	4.06 ± 2.49	5.60 ± 1.54^a^	4.02 ± 2.80	4.02 ± 2.78	< 0.001	0.232
Intensity	19.95 ± 17.61	31.02 ± 17.34^a^	20.24 ± 19.22	18.53 ± 14.66	< 0.001	0.272
SBP (mmHg)	131.67 ± 22.90	124.70 ± 20.88^a^	128.46 ± 17.91	125.02 ± 16.31^b^	0.162	0.022
DBP (mmHg)	75.30 ± 11.80	70.11 ± 11.80^a^	73.37 ± 10.18	72.36 ± 10.50	0.026	0.054
Rest heart rate (bpm)	71.58 ± 10.13	70.67 ± 9.52	74.20 ± 13.42	73.67 ± 11.63	0.857	< 0.001
6MWT (m)	540.25 ± 59.55	564.59 ± 65.64^a^	534.80 ± 72.76	532.01 ± 70.29	0.010	0.074

*Note:* Data are mean ± SD.

Abbreviations: BMI = body mass index, DBP = diastolic blood pressure, PBF = percentage body fat, SBP = systolic blood pressure, 6MWT = six‐minute walk test.

^a^ Indicates significant difference between pretest and posttest (*p* < 0.01).

^b^ Indicates significant difference between pretest and posttest (*p* < 0.05).

### 4.2 Executive Function

There were significant group‐by‐time interaction effects for all indices of the digit span test (Table 2). Follow‐up analyses showed that the scores of DSF (*p* = 0.014 and *d* = 0.38), DSB (*p* = 0.017 and *d* = 0.36), and DST (*p* = 0.001 and *d* = 0.53) increased significantly at posttest in the EX group, whereas no significant change was observed for the control group. There was also a significant group‐by‐time interaction effect for the total initiation time (TIT) of the TOL task. Follow‐up analyses showed that the TIT increased significantly at posttest in the EX group (*p* = 0.043 and *d* = 0.35), whereas no significant change was observed for the control group. No significant change or group difference was observed for other TOL indices (Table 2).

**TABLE 2:** The results of the executive function tests.

	Exergame group (*n* = 46)		Control group (*n* = 45)			F	*p*	${\eta^{2}}_{p}$
	Pre	Post	Pre	Post				
Digit span test								
DSF(points)	14.10 ± 1.59	14.42 ± 1.69^a^	14.42 ± 1.69	14.08 ± 1.91	*Time*	0.504	0.48	0.006
					*Group*	0.153	0.697	0.002
					*Time X Group*	8.773	0.004	0.09
DSB(points)	7.97 ± 2.62	8.28 ± 3.07^a^	8.28 ± 3.07	8.26 ± 2.95	*Time*	3.590	0.061	0.039
					*Group*	0.015	0.904	< 0.001
					*Time X Group*	4.049	0.047	0.047
DST(points)	22.08 ± 3.52	22.71 ± 4.25^b^	22.71 ± 4.25	22.35 ± 4.26	*Time*	3.455	0.066	0.037
					*Group*	0.061	0.806	0.001
					*Time X Group*	10.787	0.001	0.001
Tower of London task								
TMS(points)	38.43 ± 18.71	36.10 ± 18.56	39.17 ± 16.92	36.64 ± 18.55	*Time*	1.837	0.179	0.02
					*Group*	0.014	0.905	< 0.001
					*Time X Group*	0.031	0.860	< 0.001
TIT(sec)	53.96 ± 32.13	59.93 ± 34.31^a^	56.37 ± 38.20	53.28 ± 37.69	*Time*	0.812	0.37	0.009
					*Group*	0.133	0.716	0.001
					*Time X Group*	5.749	0.021	0.058
TET(sec)	257.58 ± 106.02	244.37 ± 91.30	268.90 ± 89.87	245.51 ± 101.39	*Time*	5.710	0.019	0.06
					*Group*	0.002	0.962	< 0.001
					*Time X Group*	1.123	0.292	0.012
TT(sec)	312.47 ± 115.32	305.29 ± 99.66	323.34 ± 101.45	294.26 ± 106.36	*Time*	4.733	0.032	0.050
					*Group*	0.031	0.861	< 0.001
					*Time X Group*	2.279	0.135	0.250

*Note:* Data are mean ± SD.

Abbreviations: DSB = digit span backward, DSF = digit span forward, DST = digit span total, TET = total execution time, TIT = total initiation time, TMS = total move score, TT = total time.

^a^ Indicates significant difference between pretest and posttest (*p* < 0.05).

^b^ Indicates significant difference between pretest and posttest (*p* < 0.01).

### 4.3 Heart Rate Variability

The HRV data of 14 participants were discarded due to insufficient quality of ECG data for HRV analysis. Therefore, HRV data of 77 participants (38 in the EX group and 39 in the CON group) were included in the analysis. The results showed no significant change in all HRV indices in both groups. There was also no significant group difference in HRV indices (Table 3). The results of the correlation analysis revealed that there were significant positive correlations between HFnu and DSB (*r* = 0.238 and *p* = 0.037) and between HFnu and DST (*r* = 0.225 and *p* = 0.049) (Table 4). No significant correlation was observed between other HRV indices and digit span test scores and between all HRV indices and TOL test scores.

**TABLE 3:** The results of HRV.

	Exergame group (*n* = 38)		Control group (*n* = 39)			*F*	*p*	${\eta^{2}}_{p}$
	Pre	Post	Pre	Post				
TP(ms2)	1165.28 ± 1255.18	1193.40 ± 897.22	1050.15 ± 962.37	1278.72 ± 2374.11	*Time*	3.965	0.050	0.050
LnTP	2.87 ± 0.41	2.97 ± 0.3	2.82 ± 0.43	2.89 ± 0.37	*Group*	0.668	0.416	0.009
					*Time X Group*	0.125	0.724	0.002
SDNN(ms)	34.09 ± 22.31	33.89 ± 13.59	30.56 ± 13.92	31.60 ± 18.29	*Time*	2.084	0.153	0.027
LnSDNN	1.46 ± 0.22	1.5 ± 0.16	1.43 ± 0.2	1.46 ± 0.17	*Group*	0.856	0.358	0.011
					*Time X Group*	0.033	0.856	< 0.001
RMSSD(ms)	24.96 ± 16.97	26.88 ± 18.15	25.29 ± 15.32	27.01 ± 32.10	*Time*	1.245	0.268	0.016
LnRMSSD	1.32 ± 0.24	1.36 ± 0.23	1.32 ± 0.26	1.33 ± 0.24	*Group*	0.108	0.744	0.001
					*Time X Group*	0.327	0.569	0.004
HFnu	46.63 ± 17.67	48.05 ± 23.65	41.72 ± 20.89	38.79 ± 18.08	*Time*	0.366	0.547	0.005
					*Group*	2.704	0.104	0.035
					*Time X Group*	1.744	0.191	0.023

*Note:* Data are mean ± SD. For SDNN, RMSSD, TP, the first row represents raw data (ms) and the second row represents log‐transformed data (Ln). Statistical analyses were conducted using Ln data.

Abbreviations: HFnu = high frequency normalize unit, RMSSD = the square root of the mean squared differences of successive NN intervals, SDNN = standard deviation of normal to normal, TP = total power.

**TABLE 4:** The correlation between executive function tests and HRV.

	RMSSD	SDNN	TP	HFnu
DSF	−0.013	0.047	0.024	0.078
DSB	0.113	−0.102	−0.057	0.238^a^
DST	0.079	−0.050	0.030	0.225^a^
TMS	0.026	0.022	0.023	−0.161
TIT	0.193	0.032	−0.022	0.116
TET	0.122	0.069	0.071	0.013
TT	0.159	0.074	0.073	0.037

Abbreviations: DSB = digit span backward, DSF = digit span forward, DST = digit span total, HFnu = high frequency normalize unit, RMSSD = the square root of the mean squared differences of successive NN intervals, SDNN=standard deviation of normal to normal, TET = total execution time, TIT = total initiation time, TMS = total move score, TP = total power, TT = total time.

^a^ Indicates significant correlation between executive function and HRV.

## 5 Discussion

The present study aimed to examine the effects of a 10‐week exergame training program on executive function and HRV in middle‐aged and older adults. Our results showed that the exergame training was effective in improving the performance of the digit span test and in increasing the TIT of the TOL task. However, no significant change was observed for the HRV after exergame training. Our results also showed that exergame training was effective in lowering blood pressure and improving aerobic endurance in middle‐aged and older adults.

### 5.1 Exergame Training and Executive Functions

The improved performance of the digit span test in the EX group suggests that our exergame training had positive effects on working memory (Gardner 1981). The increased total initial time of the TOL task in the EX group suggests enhanced inhibitory control after the exergame training (Culbertson 2005). Longer initial time was also shown to be associated with better planning strategy for problem solving (Berg et al. 2010; Donders et al. 2012; Chang et al. 2012; Unterrainer et al. 2004) and fewer solution errors (Ward et al. 1997). Our results are consonant with findings from previous studies that demonstrated improved executive functions after exergame training. A recent meta‐analysis included 31 randomized controlled trials examining the effects of exergame training on cognitive performance in middle‐aged to older adults (Manser, Herold, and de Bruin 2024). The results showed that exergame training was effective in improving global cognition, complex attention, and executive functions but not learning and memory or visuospatial skills. Among these studies, 20 included measures of executive functions, such as inhibition (Stroop task), working memory (digit span and N‐back task), and shifting (Trail Making Test Part B, TMT‐B). Zhao et al. (Zhao et al. 2022) conducted a randomized controlled trial that included 55 older adults (mean age 65.4 years). The exergame training group completed three 75 min training sessions per week over 12 weeks and displayed improved working memory and inhibitory control. Similarly, Adcock et al. (Adcock et al. 2019) performed three 30–40 min home‐based exergame sessions per week over 16 weeks in 31 older adults (mean age 73.9 years) and found improvements in working memory and inhibitory control. Hou et al. (Hou et al. 2023) conducted a randomized controlled trial in 128 older adults (mean age 67.9 years) with hypertension. The intervention groups (exergame and cycling) performed three 60 min training sessions per week for 16 weeks and showed improvements in working memory but not inhibitory control or cognitive flexibility when compared with the control group. No significant differences were observed between the two intervention groups. Our results add to the literature and suggest that exergame training was effective in improving working memory, inhibitory control, and planning strategy.

On the other hand, we did not observe significant changes in the other indices of the TOL task. Previous studies have suggested that the total move score was associated with better quality and efficiency of planning (Berg et al. 2002), and the total execution time and total time were shown to represent the quality of executive planning (Culbertson 2005) and the ability to monitor the process of planning (Unterrainer et al. 2004). To the best of our knowledge, the effect of exergame training on TOL performance has not been examined previously in healthy middle‐aged and older adults. The impact of exergame on the quality and efficiency of planning remains unclear and warrants further investigation.

### 5.2 Exergame Training and HRV

No significant change was observed for HRV in the present study. Our results are different from previous studies that demonstrated increased HRV after exergame training. Eggenberger et al. conducted a secondary analysis of a randomized controlled trial that included 89 healthy older adults (mean age 78.8 years). The exergame training group completed two weekly 60 min training sessions over 6 months and displayed increased HRV indices (SDNN, RMSSD, and HF power) (P. Eggenberger et al. 2020). Meanwhile, Villafina et al. performed two weekly 60 min exergame sessions over 24 weeks in 50 women with fibromyalgia (mean age 54.0 years) and found improved SDNN but not RMSSD (Villafaina et al. 2020). Our conflicting result of HRV may be the consequence of the relatively shorter training duration (10 weeks) compared to these previous studies (6 months and 24 weeks). Previous review has suggested that at least 3 months of aerobic training may be necessary to induce significant modifications in HRV (Grässler et al. 2021a, 2021b). In addition, although moderate exercise intensity is more favorable and recommended to improve executive functions (Manser, Herold, and de Bruin 2024), endurance training at higher intensities or a combination of moderate‐ and high‐intensity seems most promising to improve HRV (Grässler et al. 2021a, 2021b). In the present study, the average intensity of the exergame training was moderate intensity (50% heart rate reserve), which may not be sufficient to improve HRV. Therefore, it may be that more than 10 weeks of moderate‐high intensity exergame training is needed to enhance HRV in healthy middle‐aged and older adults. Moreover, individuals with cardiovascular and metabolic diseases are prone to have reduced HRV and are more likely to show significant improvements in HRV after exercise training as compared to healthy individuals (Grässler et al. 2021a; Kamijo et al. 2011; Pesce et al. 2009). In the present study, we excluded participants with cardiovascular disease, diabetes, or kidney disease. As a result, our participants were relatively healthier and had higher HRV compared with the general population of similar age in our country (Grässler et al. 2021b; Kuo et al. 1999). This may have contributed to the nonsignificant findings of HRV in the present study.

### 5.3 Relationship Between HRV and Executive Function

Studies that investigated the mechanisms underlying the training effect of exergame on cognitive function were scarce (Torre et al. 2022; Stojan et al. 2019). In Stojan and Voelcker‐Rehage's review, only four studies comprised neurophysiological measures, including the hemohynamic activity in the prefrontal cortex (PFC) (Eggenberger et al. 2016), event‐related oscillations over the PFC (Schättin et al. 2016), event related potentials over the fronto‐parietal cortex (Chuang et al. 2015), and brain‐derived neurotrophic factor (BDNF) (Anderson‐Hanley et al. 2012). To the best of our knowledge, only one study has investigated the relationship between HRV and executive function in an exergame intervention. Eggenberger et al. (P. Eggenberger et al. 2020) conducted a 6‐month twice‐weekly exergame training program (virtual reality video game dancing) in healthy older adults and found significant improvements in HRV indices (SDNN, RMSSD, and HF power). In addition, the HRV indices were shown to be correlated with the executive function of shifting. Similar to these findings, our results showed a small but significant correlation between HRV (HF power) and working memory. However, no significant correlation was observed between HRV and TOL performance. Our findings partially support the model of neurovisceral integration, which links the prefrontal cortex to the autonomic nervous system responsible for the sympathetic and parasympathetic control of the heart (Thayer et al. 2009). Research has shown that the regulation of cardiac autonomic function by the prefrontal cortex is vagally mediated (Thayer et al. 2009) and that high HRV, mainly high HF power, is essential for efficient cognitive performance and is related to prefrontal cortical function (Hansen, Johnsen, and Thayer 2003). Therefore, it is plausible that the improvement in executive function after exergame training may be related to the increase in vagal‐mediated HRV (HF power). Our results agree with and extend previous findings, suggesting a link between increased HRV and improved working memory after exergame training in middle‐aged and older adults. On the other hand, the relationship between HRV and other executive functions (i.e., inhibitory control and planning ability) remains unclear and requires further investigation.

### 5.4 Other Potential Mechanisms

The improvements in executive function after exergame training may also be associated with other neurophysiological mechanisms, such as increases in neural activations, BDNF, hippocampal neurogenesis, brain volume, and cerebral blood volume (Hillman, Erickson, and Kramer 2008). For instance, Anderson‐Hanley et al. reported that cybercyclers exerted higher levels of BDNF compared to a traditional cycling group after 16 weeks of intervention, suggesting a more pronounced capacity of neuroplastic adaptation in response to exergaming (Anderson‐Hanley et al. 2012). Chuang et al. applied electroencephalography and analyzed event‐related potentials (ERP) over the fronto‐parietal cortex. They demonstrated shorter ERP latencies after 12 weeks of exergame intervention (dance video games), reflecting higher processing efficiency of brain networks (Chuang et al. 2015). However, since very few exergame studies investigated neurophysiological indices, and these studies focused on different parameters (Stojan et al. 2019), the mechanisms underlying the beneficial effects of exergame training on executive function remain uncertain and warrant further investigation.

### 5.5 Limitations

The following limitations should be considered. First, more than 80% of the participants in the present study were women, which limits the generalizability of our findings to middle‐aged and older men. Although we planned to recruit both genders, we found that older men tended to have more chronic health conditions (e.g., cardiovascular disease and diabetes), and thus were more likely to be excluded from participation in this study. Second, the results for blood pressure and the digit span test should be interpreted with caution. Given small to moderate effect sizes for these measures, the magnitudes of the improvements may be modest. Third, it is essential to investigate potential factors that influence outcomes in future research. Variables such as depressed status, educational levels, smoking history, and social interaction merit thorough exploration. Finally, different exergame systems may vary substantially regarding their physical and cognitive demands (Stojan et al. 2019), and future studies may aim at comparing the effects of different types of exergames.

## 6 Conclusions

The 10‐week exergame training program was associated with enhanced executive functions of working memory, inhibitory control, and planning ability as well as improved aerobic fitness and blood pressure in middle‐aged and older adults. In addition, HRV was associated with the improvements in working memory.

## Author Contributions

I.‐H.C.: Conceptualization; supervision; writing–review and editing; funding acquisition. T.‐C.Y.: Conceptualization; formal analysis; investigation; writing–original draft. P.‐T.W.: Writing–original draft; writing–review and editing. W.‐L.W.: Supervision; writing–review and editing. Y.‐K.C.: Supervision; writing–review and editing. C.‐H.C.: Investigation. All authors read and approved the final manuscript.

## Ethics Statement

All participants were informed about the purpose, procedures, and precautions of the study before they were asked to sign and return the written Clinical Trial Informed Consent Form approved by the Kaohsiung Medical University Institutional Review Board (KMUHIRB‐SV(II)‐20150055) and were performed according to the Declaration of Helsinki. Confidentiality of participants' information was strictly maintained throughout the study.

## Consent

Written informed consent was obtained from all individuals included in this study for the publication of any identifiable information, such as images or other personal or clinical details. A copy of the written consent is available for review by the Editor of this journal.

## Conflicts of Interest

The authors declare no conflicts of interest.

## Data Availability

The datasets used and analyzed during the current study are available from the corresponding author on reasonable request.
